# Damage Detection Based on Power Dissipation Measured with PZT Sensors through the Combination of Electro-Mechanical Impedances and Guided Waves

**DOI:** 10.3390/s16050639

**Published:** 2016-05-05

**Authors:** Enrique Sevillano, Rui Sun, Ricardo Perera

**Affiliations:** Department of Structural Mechanics, Technical University of Madrid, C/ José Gutiérrez Abascal, 2, Madrid 28006, Spain; rui.sun@alumnos.upm.es (R.S.); ricardo.perera@upm.es (R.P.)

**Keywords:** structural health monitoring, PZT sensors, electro-mechanical impedance, guided wave, electro-mechanical power dissipation, non-destructive testing

## Abstract

The use of piezoelectric ceramic transducers (such as Lead-Zirconate-Titanate—PZT) has become more and more widespread for Structural Health Monitoring (SHM) applications. Among all the techniques that are based on this smart sensing solution, guided waves and electro-mechanical impedance techniques have found wider acceptance, and so more studies and experimental works can be found containing these applications. However, even though these two techniques can be considered as complementary to each other, little work can be found focused on the combination of them in order to define a new and integrated damage detection procedure. In this work, this combination of techniques has been studied by proposing a new integrated damage indicator based on Electro-Mechanical Power Dissipation (EMPD). The applicability of this proposed technique has been tested through different experimental tests, with both lab-scale and real-scale structures.

## 1. Introduction

A vast number of aerospace, civil, and mechanical infrastructures usually work under very demanding conditions, continuously subjected to both static and dynamic loads and frequently in severe environments, which can lead to either a gradual deterioration of the structure or its sudden failure. For that reason, Structural Health Monitoring (SHM) technologies have received increasing attention in recent years. Many authors have focused their research activities towards the development of these damage detection methods. In general terms, all these methods are based on the definition of different strategies that allow the structural health to be assessed with the purpose of obtaining a quantification and, if possible, the location of any damage present in the structure [1]. In order to achieve this goal, the output responses of the structure to a given input excitation are analyzed before and after damage, and therefore the definition of a baseline of the structure is first required. After collecting all the corresponding measurements, each of the damaged state measurements is compared with the defined baseline, so that every modification of the expected response of the structure can be translated into a damage indication.

Many different SHM techniques have been used over the years, based on either global or local monitoring methods [2]. Although all global approaches are usually successfully applied and well accepted [3,4,5], they are based on the lowest modes of vibration, and so are not appropriate for monitoring reduced sensing regions, which is necessary to develop an accurate and efficient early detection methodology. In this sense, a whole set of local methods has been proposed in the literature [6], many of them sharing the same drawbacks pointed out by Balla *et al.* [2], and for that reason all efforts have been placed lately on the development of “smart structures” [7,8]. Among all the proposed technologies and smart sensing solutions, transducers based on piezoelectric ceramic materials (PZT sensors in particular) are the most promising ones [9]. These materials develop an electric response over their surfaces when a certain mechanical stress is directly applied on them, according to the direct piezoelectric effect; but they are also capable of developing the reverse phenomenon, producing mechanical stresses when an electric field crosses through the piezoelectric material, according to the inverse piezoelectric effect [2]. This capability enables the material to be used both as a sensor and as an actuator simultaneously [10]. By using these smart sensors, different local damage detection techniques have been proposed, although the two most widely developed ones are those based on electro-mechanical impedance measurements and those based on guided waves.

Damage detection based on electro-mechanical impedance measurements was first developed by Liang *et al.* [11]. The electrical impedance of the PZT can be directly related to the mechanical impedance of the host structural component where the PZT transducers are attached. Since the structural mechanical impedance will be affected by the presence of structural damage, any change in the electrical impedance spectra might be an indication of a change in structural integrity [12,13,14]. On the other hand, guided waves were used for non-destructive testing (NDT) purposes for the first time by Woriton in [15]. Typically, an array of sensors is attached to the structure and used to inject a pulse of guided-wave energy. The same array is then used to record the reflected signals, and the position of these signals in the time domain can be easily related to the position of features (e.g., flanges, welds) in the structure. Any signal that cannot be related to a known feature is assumed to be a defect. As the number of features becomes higher, their associated signals merge together obscuring the reflected signals from damage. A good review of different applications of this technique can be found in [16].

Hence, these two techniques have been widely proven to have a great potential for SHM purposes. However, few new approaches have been proposed so far [17,18], at least to the knowledge of the authors, that combine the advantages of each of them. Furthermore, more complicated structures need to be tested. In that sense, a new approach is proposed in this paper in order to combine impedance signatures with guided waves to evaluate the presence of different typologies of damage in different structures. A new damage indicator, Electro-Mechanical Power Dissipation (EMPD), is firstly introduced in this paper, and then tested using two controlled lab-scale experiments for it as well as a real-scale complex structure.

## 2. Electro-Mechanical Power Dissipation as a New Damage Indicator

### 2.1. Basis of Piezoelectric Sensing for SHM

Lead-zirconate-titanate (PZT) materials have been subject to increasing interest in recent years due to their light weight and variety of shapes and sizes [10,19,20,21], besides their wide range of applications in many research fields. In particular, they provide very promising sensing and monitoring solutions, and so these smart materials have been widely applied for Structural Health Monitoring purposes. With these smart sensors, the electrical impedance can be measured at high frequency ranges so that the wavelength of the excited motion is small and sensitive enough to detect local damage [13,22]. This constitutes the basis of the so-called Electro-Mechanical Impedance Method (EMI), whose core idea is that the presence of damage in the structure under study will affect its mechanical properties and, in turn, the electro-mechanical properties of the PZT patch, which can be directly measured by means of an impedance analyzer. The coupled relationship between the electrical and mechanical impedances was first introduced by Liang *et al.* [11] as follows:

(1) $$Y\left(\omega \right)=\frac{I_{0}}{V_{i}}=j\omega a\left[{\bar{\varepsilon }}_{33}^{T}-\frac{Z_{s}\left(\omega \right)}{Z_{s}\left(\omega \right)+Z_{a}\left(\omega \right)}d_{3x}^{2}{\hat{Y}}_{xx}^{E}\right]$$

where Y(ω) is the electrical admittance (inverse of impedance), $V_{i}$ is the input voltage to the PZT actuator; $I_{0}$ the output current from the PZT; a, ${\bar{\varepsilon }}_{33}^{T}$, $d_{3x}^{2}$, and ${\hat{Y}}_{xx}^{E}$ are the geometry constant, complex dielectric constant, piezoelectric coupling constant, and complex Young’s modulus of the PZT in a state without stresses, respectively; $Z_{s}\left(\omega \right)$ and $Z_{a}\left(\omega \right)$ are the impedances of the structure and the PZT actuator, respectively. As has been proved (Park *et al.* [23]), the real part is less sensitive to ambient temperature change, compared to the imaginary part [18]. Furthermore, the imaginary part is mostly related with the capacitance of the piezoelectric transducer. This makes it harder to extract the mechanical data from the imaginary component, making it is less suitable for damage identification purposes. Because of this, the real part of the impedance is usually used for the EMI method, and so this has been done in this paper.

However, piezoelectric materials offer wider possibilities rather than those related to the EMI method. Among all of them, guided elastic waves are increasingly used in SHM applications [24]. These waves are called guided waves because the propagation of the elastic wave is confined by the boundaries of the structure itself [25,26,27], as will be the case of the experiments carried out in this work. Because of the confined nature of these waves, it has been demonstrated that they can travel relatively long distances through the material, achieving a long sensing range for some damage detection applications [18,28,29]. Hence, this technique allows a wider sensing region than the EMI method. However, in most of applications found in the literature, the guided waves technique is often limited to simple structures [10,18,26], basically lab-scale structures made of aluminium and steel, or some composite structures in the most complex cases. In this work, more complex structures will be studied in order to explore the capabilities of this technique.

More details about this technique can be found in [24,27].

### 2.2. Definition of Electro-Mechanical Power Dissipation

One of the most interesting features of PZT sensors is that they constitute the key element of two of the most studied and successful NDT techniques nowadays: the EMI method and guided waves. However, even though these two methodologies have demonstrated great performance, few efforts have been made so far, at least to the knowledge of the authors, in the development of an integrated method that can combine both impedance signatures and guided waves in order to take advantage of the whole potential of these smart sensors. One of the few studies addressing this challenge is found in [18], where An *et al.* proposed an integrated damage diagnosis procedure based on those two different signals. However, in that study, impedance signatures and guided waves were treated separately so that two different damage indicators were obtained, both of them being weighted and accordingly combined afterwards in order to achieve a unique and integrated damage indicator. In this sense, the work presented in this paper aims to achieve a more integrated combination of the physical properties of both kinds of signals into a single one. By doing this, the necessity of weighting different damage indicators would be avoided, and thus the damage identification might be done with less *a priori* information.

To perform an advantageous combination of the information yielded by impedance signatures and guided waves, a reinterpretation of both should be carried out. On the one hand, the impedance curves used in this work will provide the evolution of the real part of the coupled electro-mechanical impedance of both the sensor and the host structure within a frequency range (in this case, from 10 to 100 kHz) and in a small area around each PZT sensor. On the other hand, with the guided waves, a signal in time domain, with a particular frequency and maximum amplitude, will be sent from one transmitter sensor to a receiver sensor, measuring the distortion between the voltage sent and received. In a simplistic way, an analogy can be established between the system PZT-host structure and a simple electrical system by means of including the impedance and voltage data measured in the traditional Ohm’s Law, and assuming as negligible all the possible mathematical or physical inaccuracies of this approach. By doing this, and by assuming that no information about the current is available for any of the experiments carried out in this work (only voltage and electro-mechanical impedances are measured), the plane definition of power can be rewritten as follows

(2) $$P=V\cdot I=\frac{V_{m}^{2}}{R}=\frac{V_{m}{\left(V_{e},t\right)}^{2}}{R}$$

where $V_{m}\left(V_{e},t\right)$ is the instantaneous voltage of the guided wave measured at the second sensor, which depends on the time as well as on the voltage of excitation *V_e_*, both real values at a particular frequency $\omega_{gw}$; *R* represents just the real part of the ideal impedance (resistance). The dependence between *V_m_* and *V_e_* is just considered in this work regarding the amplitude and shape of the guided waves. Hence, if any of these parameters were changed for the voltage of excitation, it would have a direct impact on the corresponding parameters of the measured voltage, which would happen within a given time frame.

From Equation (2), an instantaneous Electro-Mechanical Power Dissipation (EMPD) is defined from the impedance as follows

(3) EMPD(t)=Re(ifft(fft(Vm(Ve,t))2Re(Z¯(ω)|ωgw)))

where $\bar{Z}\left(\omega \right)$ is the average of the electro-mechanical impedances measured at the frequency $\omega_{gw}$ obtained from the PZT transmitter and receiver sensors; *Re* indicates that only the real part of the electro-mechanical impedance is considered, fft denotes the Fast Fourier Transform, and ifft the Inverse Fast Fourier Transform. As the Inverse Fast Fourier Transform provides a set of complex values, just the real part of the EMPD will be used in this damage detection procedure.

At this point, it is worth clarifying that several assumptions have been made in order to define this time-dependent variable (EMPD):

- The guided waves used in this work are sent and received at a particular frequency, while the impedance signatures are obtained by performing a sweep in the frequency domain. This implies that, after expressing both signals in the frequency domain, the electro-mechanical impedance will show a different amplitude at each frequency. However, after applying the corresponding filter, just one significant non-zero amplitude will be found for the measured voltage in the frequency domain. For this reason, just the corresponding value at this frequency is taken from the electro-mechanical signal.
- As it is not the purpose of this paper to provide a rigorous mathematical explanation about the analogy between the EMPD and the one measured on a real electric circuit, the authors have not considered any phase dependency between the voltage and the current, which has been possible due to the fact that absolute magnitudes have been used in Equations (2) and (3).

A similar approach, although in a very different application, can be found at [30], where power dissipation is determined by measuring all the changes in electric impedances that a dither piezo presents to an oscillator. Although the theory behind the approach presented in [30] is certainly more complex, it may be of interest to those readers wishing for a more detailed and rigorous theoretical explanation.

### 2.3. Damage Detection Procedure

**Figure 1:**
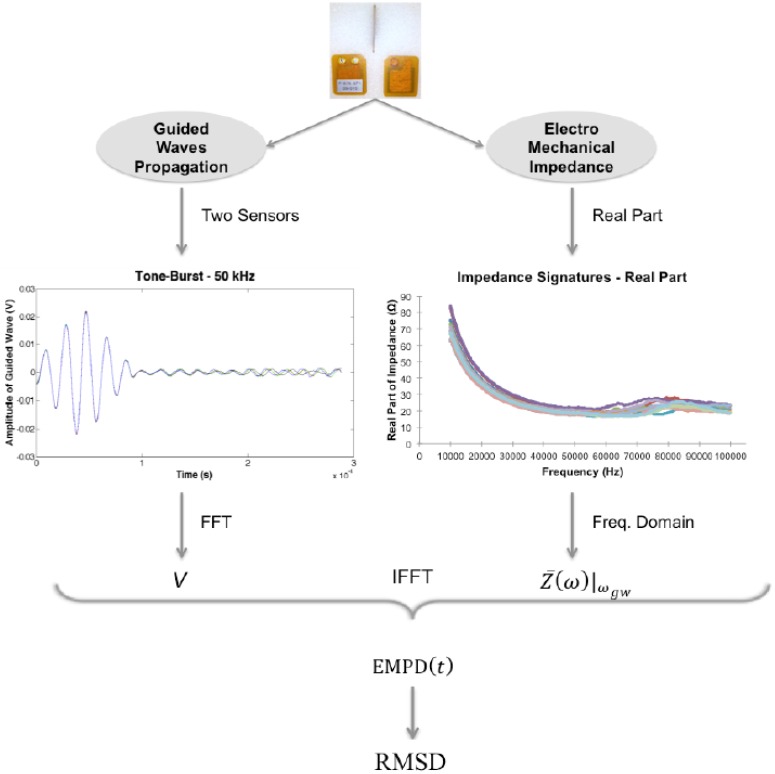
Overall procedure to extract the Damage Indicator based on Electro-Mechanical Power Dissipation.

The overall procedure of the proposed integrated damage detection technique is illustrated in Figure 1. This process starts by measuring, separately, the impedance signatures and the guided waves for a baseline stage of the structure, if possible, in a healthy state. Thanks to the combination of both signals at a particular frequency, it is then possible to obtain EMPD in the time domain for different damage conditions or stages. All possible deviations in the EMPD signal, when compared with the baseline stage, are finally interpreted as a damage increment through the definition of a damage indicator. The detailed procedure of this proposed techniques can be summarized as follows:

(a) Collection of baseline impedance signals and guided waves. In the case of the electro-mechanical impedance, one signature is obtained per PZT sensor embedded in the host structure. In order to enhance the accuracy of the process, several measurements can be taken in order to work in the subsequent stages with the average of all those measurements. Once the impedance signatures are stored, guided waves will be measured, obtaining two different signals between every two sensors: one sent from the first sensor and measured at the second one, and vice versa.
(b) All experimental procedures imply the presence of environmental noise, and given that these sets of measurements are taken at high frequencies, the following step is to clean each signal from undesired noises. The influence of the noise on the electro-mechanical impedance is already minimized thanks to the impedance analyzer used, which employs a measuring technique that calculates the average between a number of neighbouring sample points, but a bandpass filter (which depends on the particular application) is needed for the guided wave signals.
(c) To compute EMPD, the Fast Fourier Transform (FFT) is applied to all guided wave signals in order to obtain their corresponding values in the frequency domain. As each of these signals is sent and measured at a particular frequency, a single point would be expected in the frequency domain. However, there is still some negligible noise present after the filtering process, which is going to reveal some more frequencies after the FFT is applied. The corresponding value of the electro-mechanical impedance at that frequency must be selected, so that the Electro-Mechanical Power can be defined using an extension of Equation (2). As only one impedance signature is available per sensor, the value considered in this case has been the average of the impedances measured at the corresponding two consecutive sensors used to measure each guided wave.
(d) The Inverse Fast Fourier Transform (IFFT) is applied in order to obtain the expression of the EMPD in time domain. The signal obtained in this step will be used as a baseline in the subsequent iteration, so only the appearance of new damage with respect to the baseline stage should be reflected by the damage index.
(e) Repeat steps (a) to (d) for each of the studied damage cases of each experiment, so that the corresponding EMPD signatures can be obtained for each health condition of the structure.
(f) Once the EMPD is obtained for each of the damage scenarios, the following step is to define an appropriate damage indicator that allows the analysis of the health condition of the corresponding structure. The root mean square deviation (RMSD) is the most commonly used indicator to assess damage [20,31,32], and is computed from the difference in the EMPD value at each timepoint as
  (4) $$RMSD\left(\%\right)=\sqrt{\frac{\sum_{i=1}^{n}{\left[EMPD_{0}\left(t_{i}\right)-EMPD_{1}\left(t_{i}\right)\right]}^{2}}{\sum_{i=1}^{n}EMPD_{0}{\left(t_{i}\right)}^{2}}}\cdot 100$$
  where $EMPD_{0}\left(t_{i}\right)$ is the EM power dissipation of the PZT measured at a previous stage, which might agree with the healthy condition of the structure; $EMPD_{1}\left(t_{i}\right)$ is the corresponding value at a subsequent stage, which might agree with a post-damage stage, at the *ith* timepoint; *n* is the number of timepoints. For the RMSD index, the larger the difference between the baseline reading and the subsequent reading, the greater the value of the index denoting changes in structural dynamic properties which can be due to damage.

## 3. Experimental Study for Lab-Scale Structures

In order to check the proposed methodology, some previous experimental tests were carried out. These included two lab-scale structures such as a simple bolt-jointed aluminium beam and an FRP-strengthened concrete specimen. These two tests were considered as the basis for a third experimental study, which consisted in the identification of debonding on a full-scale reinforced concrete beam strengthened with an FRP strip.

At this point, it is important to remark on the influence of the environmental conditions on the measurements taken to build the EMPD signals. Regarding the impedance signatures, some slight variations were observed between consecutive measurements, as many authors have reported in the literature referenced in this work. In order to prevent this variability affecting the final damage predictions, averaging has been made in all the experiments performed in this work, following the recommendations found in the literature. This minimizes the influence of these variations or deviations in the whole damage prediction procedure. Exactly the same problem was observed when measuring guided waves at each sensor. Once again, averaging was the adopted solution, as explained below at each of the experiments. However, in this case, some outliers were also found for some measured signals. As the number of outliers found were small enough (no more than five times in the whole procedure), and as each signal was checked *in situ* after measured, the authors decided to neglect these signals and just take new measurements.

On the other hand, temperature changes might affect measured characteristics, as some authors have addressed in the literature [18,33,34]. However, most of these studies including temperature effects allow large temperature range variations, e.g., between −20 °C and 40 °C [18], which is not the intention of the work presented in this paper. Each of the lab-scale experiments were performed in approximately the same conditions, which were in a room maintained at 24°C during the whole experiment. The authors estimate that temperature variations no larger than 2 °C could be found in this room. Regarding the experiment real-scale specimen, the same conditions were maintained, this time with a temperature of 27 °C and with the same estimated temperature deviations. For this reason, the authors did not consider temperature effects as a source of uncertainty for the results presented in this section.

### 3.1. Bolt Loosening Detection in an Aluminium Lap Joint

**Figure 2:**
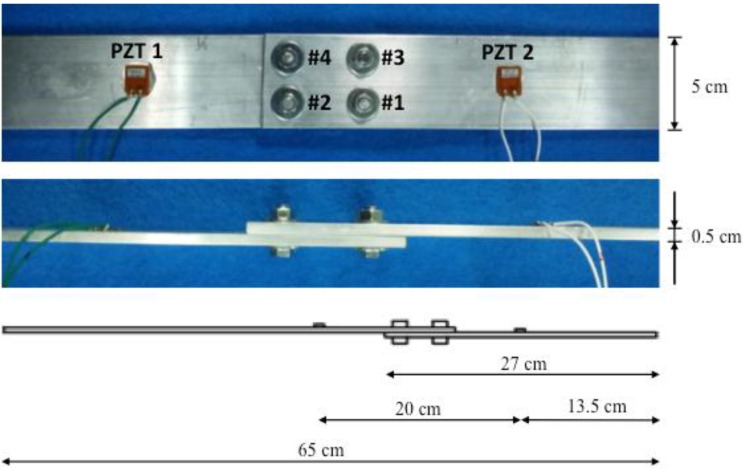
Bolt-jointed aluminium beam with two PZT transducers.

The first test was carried out on a lab-scale bolt jointed aluminium specimen consisting of two beams, the first one with dimensions of 45×5×0.5 cm^3^ and the second one with dimensions of 27×5×0.5 cm^3^, connected by four bolts with a diameter of 10 mm (Figure 2). Two identical P-876 DuraAct Patch Transducers [35] were bonded to both sides of the lap joint at a distance of 20 cm between them by using an epoxy adhesive. The purpose of this test was to evaluate the ability of the proposed approach to detect the damage due to loose bolts by inducing the following four stages or damage scenarios in the analyzed specimen: (a) No damage (D0), for which five different and independent measures were taken and used as raw data (D1 to D5); (b) Damage 1 (D1): Loose bolt #3 by one half-cycle; (c) Damage 2 (D2): Loose bolt #3 by another half-cycle; (d) Damage 3 (D3): Loose bolt #2 by one-half-cycle. It is important to remark here that all the necessary measurements were taken one after another for each subsequent damage condition. In this way, for the D0 condition, both impedances and guided waves were initially measured. Once these measurements were collected, damage D1 was induced and the new stage was measured and so on.

**Figure 3:**
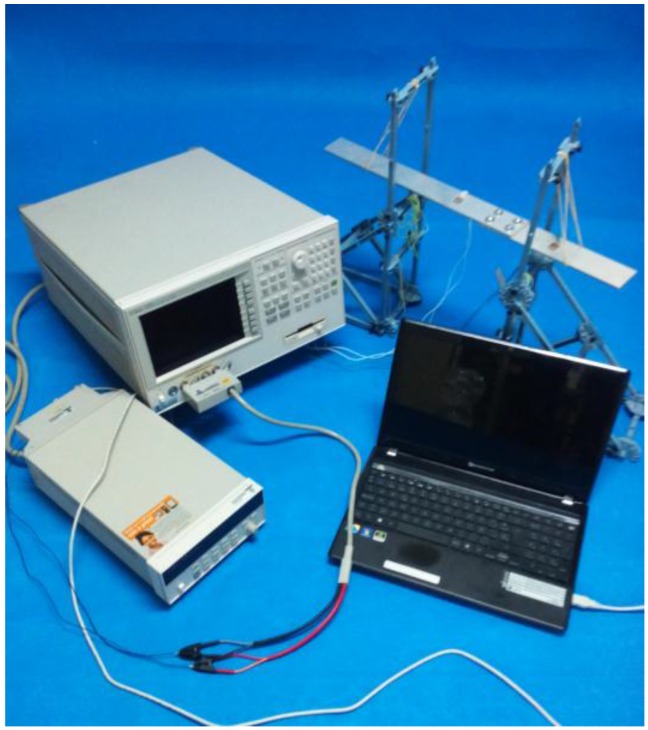
Experimental setup for impedance measurements on the bolt-jointed aluminium beam.

The two PZT sensors were individually connected to two different channels of the 3499B multiplexor, which was used to make a multiple connection between the HP 4192A Impedance Analyzer and the PZT sensors. Both, the multiplexor and the impedance analyzer were from Agilent Technologies. To measure the impedances at each sensor, a sinusoidal sweep voltage with a 1 volt amplitude was applied to the PZT sensors over a frequency range of between 10 kHz and 100 kHz. Five impedance signatures were measured for each damage case, so the raw data were obtained by averaging the responses of five measurements. Figure 3 shows the overall experimental setup for the impedance measurements.

**Figure 4:**
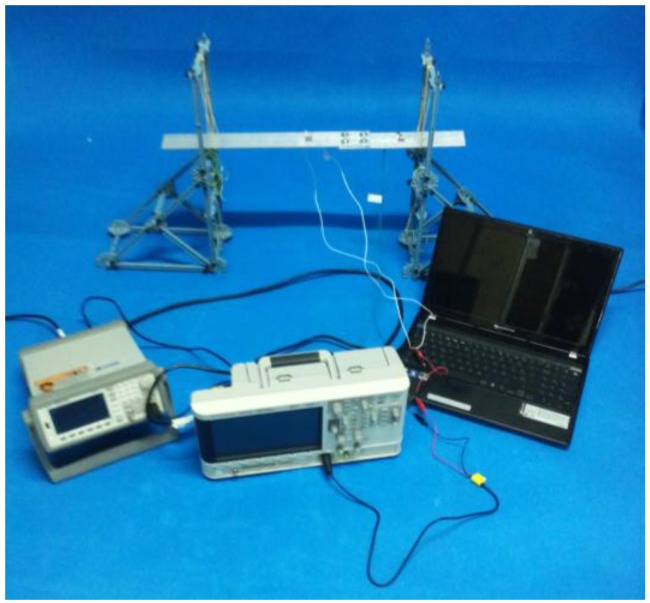
Experimental setup for guided waves measurements on the bolt-jointed aluminium beam.

Once all the impedance data were collected at each damage step, each sensor was afterwards and alternatively connected to the guided waves system, which consists on the following components: a waveform generator that sends the selected waveform to the structure in the form of an input excitation on the first sensor, an oscilloscope to register the response of the structure on the second sensor, and a conventional laptop controlling both of them (Figure 4). Both the waveform generator and the oscilloscope were acquired from Agilent Technologies. In this test, the same 10-cycle tone-burst was sent in all cases at 50 kHz with a peak amplitude of 10 volts, and five time signals were measured and averaged. Finally, a bandpass filter with 1 kHz and 500 kHz low and high cutoff frequencies was used to improve the quality of the guided signals.

The frequency used for the guided waves, which is the same as that used to generate the EMPD signatures, has been selected based on previous results, both published by the authors of this paper and found in the literature as well. Usually, guided waves work much better with high frequencies, even higher than those used by the EMI method [18], but it has also been shown in the literature that the amplitude of the impedance signatures decreases with the frequency. Thus, a trade-off must be found so that both guided waves and impedance signatures can be equally significant in the calculation of the EMPD, and so 50 kHz was set in order to test the methodology proposed in this work.

**Figure 5:**
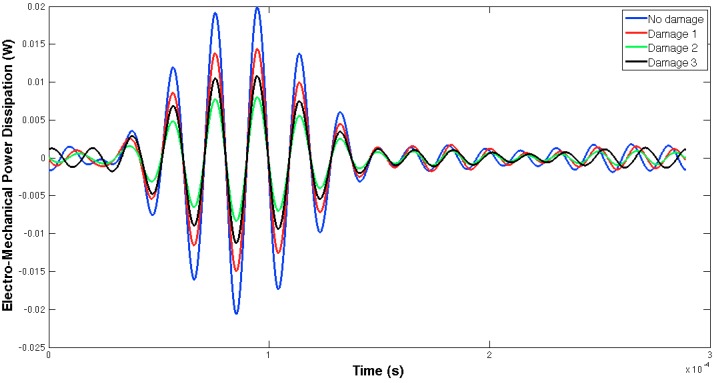
EMPD at 50 kHz. Tone-burst sent from sensor 1 to sensor 2.

**Figure 6:**
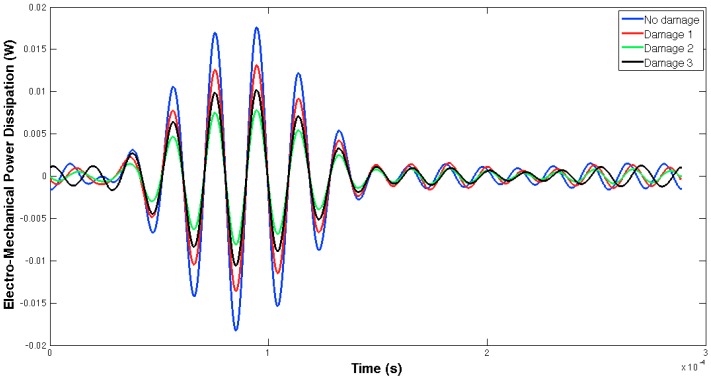
EMPD at 50 kHz. Tone-burst sent from sensor 2 to sensor 1.

**Figure 7:**
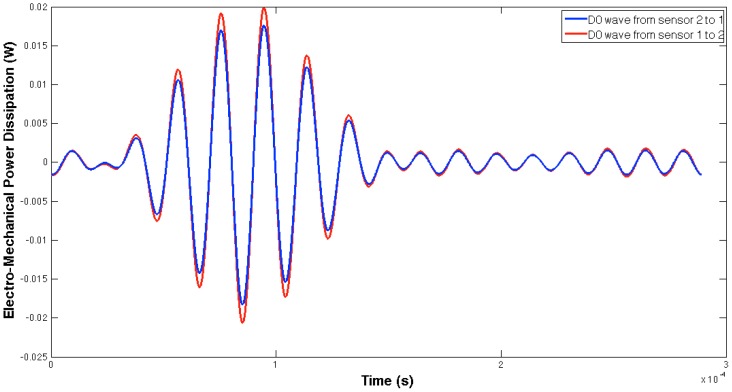
Differences in the EMPD in the aluminium lap-joint for damage D0 when the wave travels opposite directions.

Figure 5 and Figure 6 show, respectively, the EMPD obtained when the guided tone-burst is sent from sensor 1 to sensor 2, and vice versa. As can be immediately verified from these figures, there is a visible variation in the EMPD every time new damage is induced in the bolt-jointed aluminium beam, which indicates that the EMPD is sensitive to the presence of, at least, this kind of damage in this sort of lab-scale structures. Furthermore, this variation in the EMPD, which appears to be affecting the amplitude of the measured signal, is not exactly the same in both figures, since it slightly depends on the wave direction. This conclusion might not be too obvious to extract from these figures, but it is quite easy to check by a simple comparison between the corresponding arrays of output data at each damage level. An example of this is shown in Figure 7 for damage D0 and the wave travelling in both directions. Nevertheless, further information might be extracted if Equation (4) is used to translate these data into a damage indicator.

**Figure 8:**
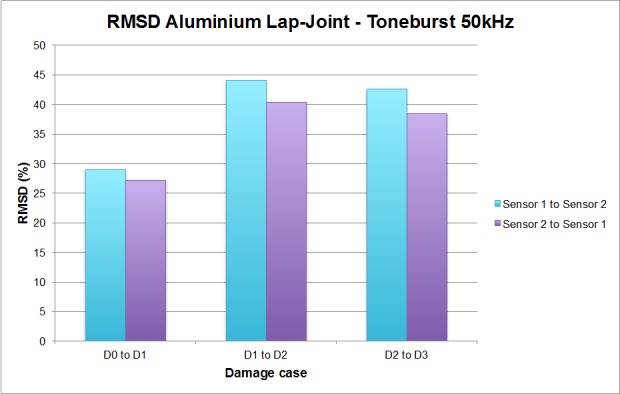
RMDS values for the bolt-jointed aluminium beam.

Figure 8 shows the computed values of the RMSD corresponding to the two previous figures, one value per damage stage and wave direction. As commented above, this figure shows a clear sensitivity to the appearance of damage in all cases. Nevertheless, a clear difference cannot be found between single and multiple damage scenarios. In the two single damage scenarios, D1 and D2, the RMSD is higher when the tone-burst travels towards sensor 2 (1.78% higher for D1 and 3.7% higher for D2, where damage is more severe), and this sensor turns out to be the one closer to the induced damage (bolt #3). From this statement it is tempting to extract the general conclusion that the variation in the EMPD will be higher when the damage is closer to the sensor in which the tone-burst is received, and this might work in that way for all single damage scenarios, but it is certainly not true when addressing the case of a multiple damage scenario (D3). In this case, even though there is new damage induced close to sensor 1 (bolt #2), the RMSD calculated in the other direction is still higher, which clearly contradicts the conclusions from the single damage scenario. However, this might make sense given that the damage severity at bolt #3 is higher than the new damage at bolt #2. Also, considering the proximity between the two bolts makes the process more difficult.

**Figure 9:**
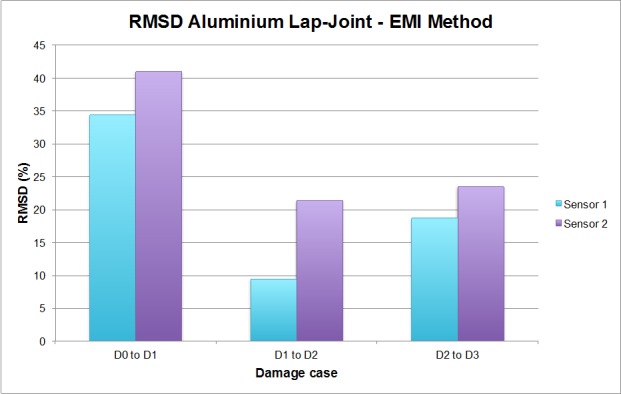
RMDS values for the bolt-jointed aluminium beam corresponding to the EMI method.

These damage predictions can be now compared to the ones obtained from the traditional EMI method, which are shown in Figure 9.

By means of this comparison, it can be stated that the EMPD is more sensitive to the appearance of new damages (D2 and D3) than the traditional EMI, given that the RMSD values are significantly lower after the first damage scenario when using the EMI method instead of the EMPD, even though additional damage was induced in the specimen. Therefore, it is possible to obtain a better estimation of the severity of the total damage in the specimen by using the EMPD signatures, making them more sensitive to the presence of damage. However, attending to the results after damages D1 and D2, it has to be pointed out that the estimation of damage localization is not really improved with respect to the EMI method, particularly in the case of damage D2, since the differences in the RMSD values between sensors are larger for the EMI method. On the other hand, both methods give an incorrect localization for damage D3, which is the most severe and complex in the specimen.

Thus, with this first experiment, the sensitivity of the EMPD to the presence of damage, in both single and multiple scenarios, has been demonstrated to be higher than with the EMI method. Furthermore, the use of the EMPD seems to be promising when localizing damage in single damage scenarios, although accurate damage localization is apparently not possible when multiple damages are induced in the structure. Actually, at least in this experiment, the localization does not improve the results obtained with the EMI method. In order to clarify this point and to test the real potential of the proposed methodology, more experiments have been carried out in this work.

### 3.2. Induced Debonding on an FRP-Strengthened Concrete Specimen

**Figure 10:**
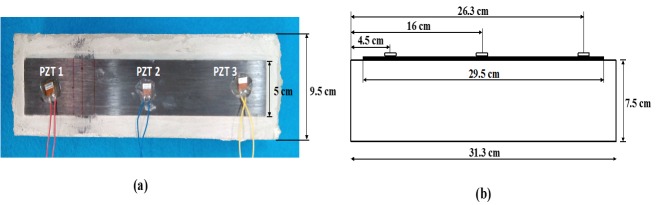
FRP-strengthened concrete specimen with three PZT transducers.

The second test was carried out over a concrete specimen with dimensions 31.3×9.5×7.5 cm^3^ which was strengthened with an external FRP strip, with dimensions 29.5×5×0.18 cm^3^, bonded by using an epoxy resin adhesive. Three identical P-876 DuraAct Patch Transducers from Piceramics [35] with the thickness of 0.5 mm were symmetrically bonded along the length of the specimen using the same epoxy adhesive as before (Figure 10). The overall experimental setups for the measurements are the same as the ones shown in Figure 3 and Figure 4, and the same measurement conditions were imposed on both the impedance signatures and the guided waves.

**Figure 11:**
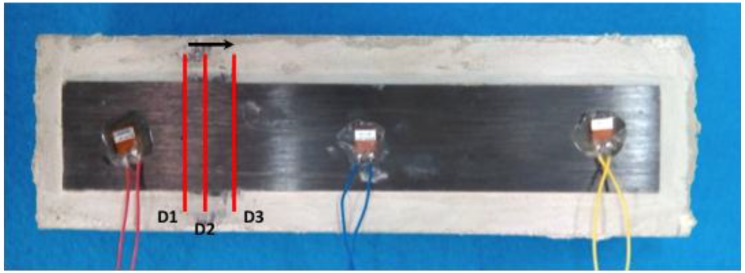
Damage scenario for an FRP-strengthened concrete specimen (I).

**Figure 12:**
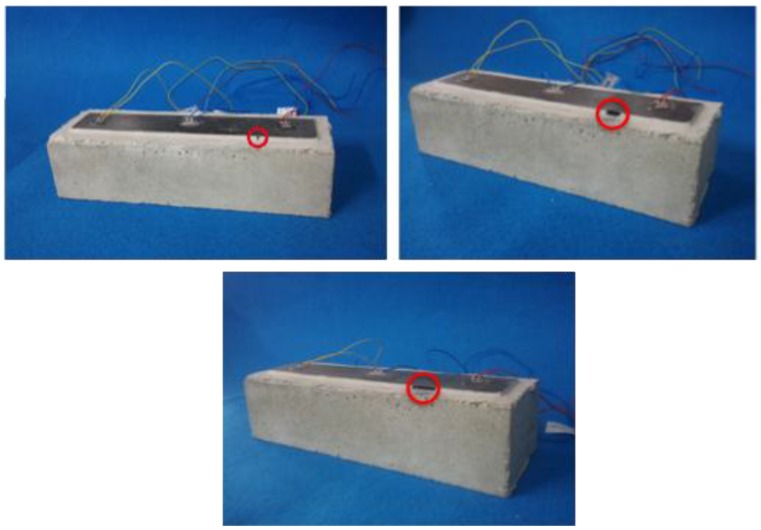
Damage scenario for an FRP-strengthened concrete specimen (II).

The purpose of this final test was to simulate, over a lab-scale concrete specimen, the debonding failure mode that usually comes up in real structures strengthened with FRP strips. In order to detect that failure through the application of the proposed method, the following four stages or damage scenarios were induced (all by means of holes drilled in the resin adhesive) in the analyzed specimen: (a) No damage (D0); (b) Damage 1 (D1): a 5 mm debonding located 7.25 cm away from the left end of the specimen; (c) Damage 2 (D2): amplification of the debonding practised in the previous stage towards the PZT number 2, up to a total debonding length of 1 cm; (d) Damage 3 (D3): repetition of the previous step up to a total debonding length of 2.5 cm (Figure 11 and Figure 12).

**Figure 13:**
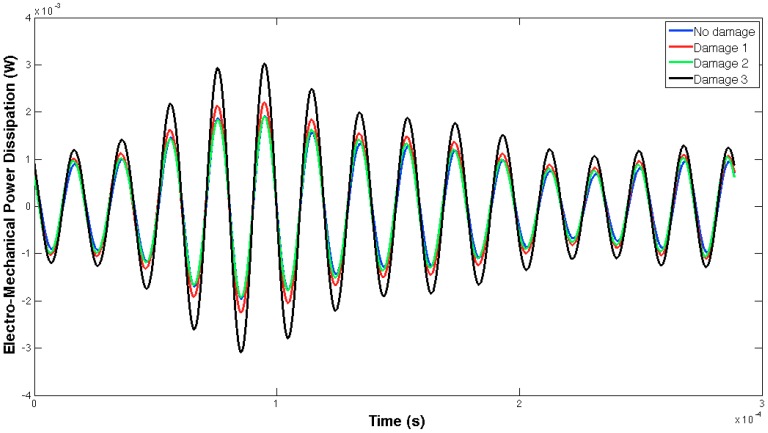
EMPD at 50 kHz. Tone-burst sent from sensor 1 to sensor 2.

**Figure 14:**
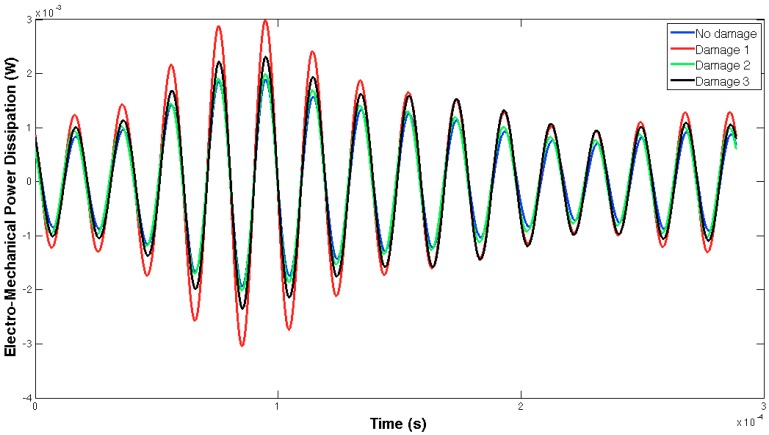
EMPD at 50 kHz. Tone-burst sent from sensor 2 to sensor 1.

**Figure 15:**
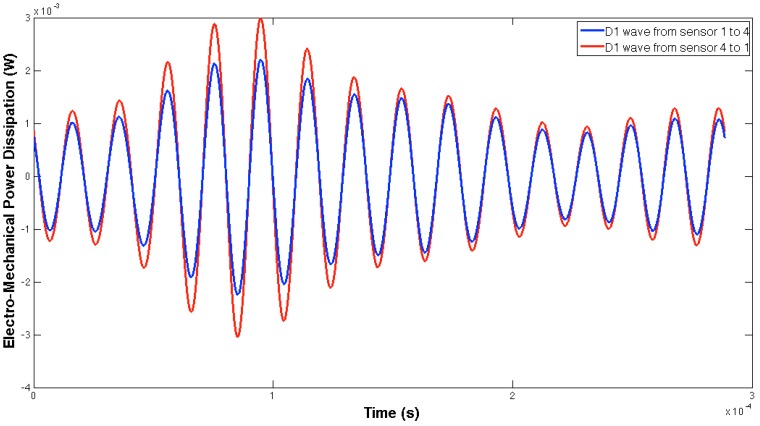
Differences in the EMPD in the concrete specimen for damage D1 when the wave travels opposite directions.

In this case, both the structural properties and boundaries, as well as the nature of the damage, are totally different from those used in the previous example, so different EMPD results are expected. These differences can actually be found in Figure 13 and Figure 14, where not only the shape of the EMPD is different from the previous example, but also the EMPD values depending on the wave direction, which is much clearer than in the previous example (Figure 15).

**Figure 16:**
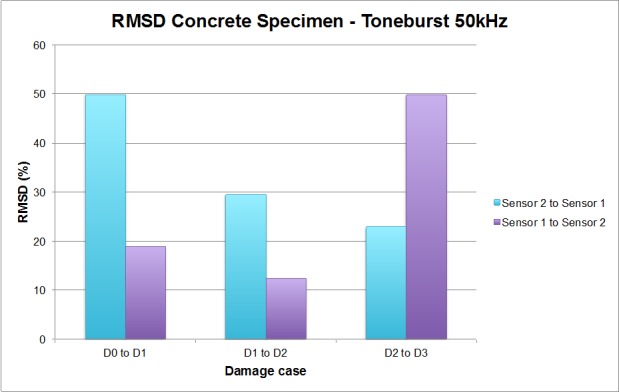
RMSD values for the concrete specimen.

As in the previous example, these differences encountered in the EMPD can be evaluated though the RMSD index in order to obtain conclusions about damage presence in the concrete specimen. These values are, again, calculated once per damage stage and wave direction, as shown in Figure 16.

In this test, a continuous damage scenario is studied. It cannot be considered either a single or multiple damage scenario in the same sense as in the previous example, since it is not possible to specify the location of each damage at a particular position. From the initial location of damage (case D1) defined at the concrete-FRP interface, an extension towards sensor 2 was practised for the other two damage cases, D2 and D3, with the purpose of increasing the severity and extension of the damage. As seen in Figure 16, the RMSD for D1 is higher when the guided wave travels towards sensor 1 (49.84% *vs.* 18.87%), which means, according to the conclusions extracted in the previous example, that the damage D1 is close to this sensor, as it actually is (Figure 11). Furthermore, from the noticeable difference in the RMSD values for this case, given that if the guided wave travels in the opposite direction the value is 30.97% lower, it is reasonable to say that the distance between the damage position and sensor 1 is much lower than that between the damage and sensor 2, which turns out to be true.

For the D2 damage case, whose severity is higher than in the D1 scenario, again the RMSD is higher when the guided wave travels towards sensor 1, with a difference of 17.08% this time. According to this, it is again concluded that the damage is located closer to sensor 1. However, the RMSD value is quite smaller than that corresponding to D1, when a similar value might be expected. This reduction, from the experience of the authors, is quite normal given that the first hole made in the adhesive implies bigger alterations of the mechanical properties of the specimen than those induced by the second one. In a real-scale specimen, a hole of those characteristics would be more than sufficient to initiate the failure mechanism due to debonding propagation. Furthermore, it should not be forgotten that the RMSD values are computed between two consecutive stages. Therefore, this parameter should only reflect any change between one stage and the consecutive one, so consistently higher RMSD values cannot be expected for the same sensor at different damage stages, since damage is being extended along the specimen in the opposite direction (from sensor 1 to sensor 2 in this case).

Finally, in the D3 damage case, the RMSD values are much higher when the EMPD is calculated from the measurements at sensor 2, which, according to the reasoning proposed in this paper, would erroneously mean that this damage is much closer to sensor 2 this time, given that the maximum extent of this hole reached the midpoint between the two sensors. Nevertheless, considering just the RMSD values calculated when the guided wave travels towards sensor 1, it is quite clear that the value corresponding to each of the damage cases is lower each time, which suggests that the new debonding between the concrete and the FRP is further each time from this sensor, as can be verified from Figure 12. Taking into account again the expression defined in Equation (4) for the RMSD indicator, the values computed between D2 and D3 stages should only capture the new debonding practiced at the interface from the D2 stage.

**Figure 17:**
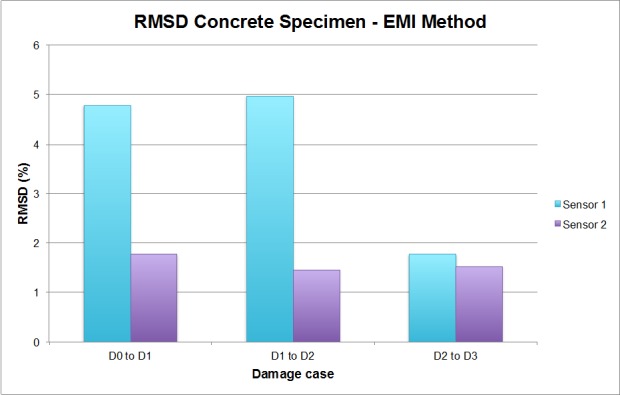
RMSD values for the concrete specimen corresponding to the EMI method.

By making a comparison, as in the previous example, with the RMSD obtained by applying the traditional EMI method, shown in Figure 17, it is even clearer in this case that the proposed methodology is quite more sensitive to damage than the traditional EMI method. Furthermore, it might be true that the proposed methodology fails in the localization of damage D3, but the values of the RMSD shown in Figure 16 are more representative of the real damage present in the concrete specimen after inducing D3, which is actually the biggest damage induced in this example (see Figure 12). In comparison to the damage found by the EMPD, the values obtained with the traditional EMI can be considered negligible, so even the conclusions about damage location cannot be taken into account. In terms of RMSD differences between sensors 1 and 2 at each damage stage, it is clear as well that damage conclusions are much more evident by using the EMPD.

Again, this methodology shows promising results when dealing with concentrated damages (D1), even despite the complexity of both the structural system and the nature of the damage. However, the location of the damage is not equally good when the extent of damage increases (in the case of the debonding, although accurate results have been presented for D2), or when it is affecting different sensing regions along the beam (bolt-jointed beam), although those are scenarios that should not be reached in any real structure if a good early detection system is properly developed and deployed. Nevertheless, the sensitivity of the EMPD to damage appearance is clearly demonstrated, even for more complex specimens.

## 4. Debonding Assessment in a Real-Scale RC Beam Externally Strengthened with FRP

A third experimental study was carried out to test the feasibility of the proposed approach when applied to a real-scale complex structure, such as that corresponding to a reinforced concrete beam externally strengthened with an FRP strip. The failure mode of this kind of structure is critical because it is usually due to the sudden and brittle debonding of the FRP reinforcement originating from an intermediate flexural crack [36,37,38]. Detection of debonding in its initial stage is essential to prevent future failure, which might be catastrophic.

### 4.1. Test Description

**Figure 18:**
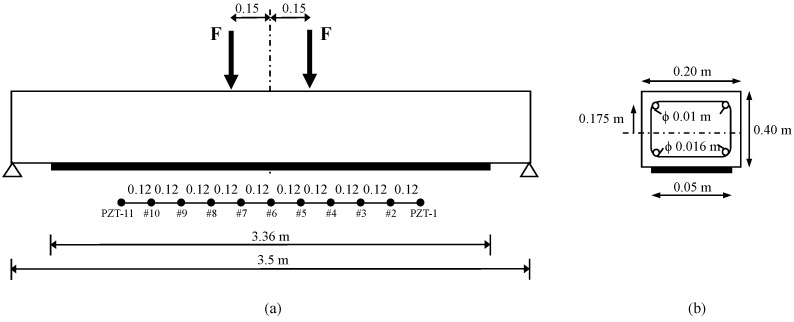
(**a**) Geometry, loading scheme, and sensor location map for the RC beam; (**b**) Cross-section of the beam.

The geometric dimensions and the reinforcement layout in the sections are illustrated in Figure 18. The identified material properties are: (a) for concrete, the elastic moduli, compressive strength, mass density, and Poisson coefficient were taken to be E=24,858 MPa, $f_{c}=24.64\text{ MPa}$, $\rho =\;2350\;\mathrm{kg}/m^{3}$, and υ=0.2, respectively; (b) for steel reinforcement, the elastic moduli, elastic limit, mass density, and Poisson coefficient were taken to be E=210,000 MPa, $f_{y}=510\text{ MPa}$, $\rho =\;7850\;\mathrm{kg}/m^{3}$ and υ=0.3, respectively; (c) for FRP external reinforcement, the elastic moduli and Poisson coefficient were taken to be E=150,000 MPa and υ=0.35, respectively, with a thickness of $e_{FRP}=1.4\;\mathrm{mm}$; (d) for adhesive, the shear moduli was assumed to be G=4300 MPa and the thickness $e_{ad}=3.45\;\mathrm{mm}$.

**Figure 19:**
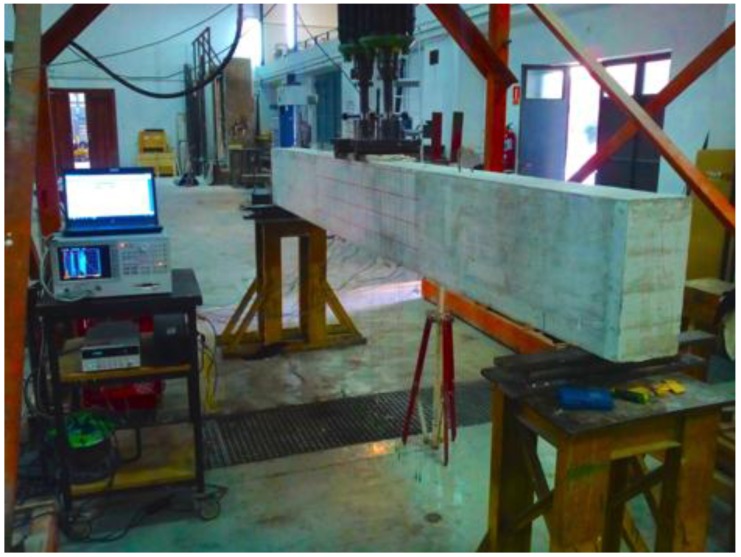
Experimental setup for the reinforced concrete beam externally strengthened with an FRP strip.

In the test programme performed, the strengthened beam was subjected to a series of four-point increasing static load tests with the purpose of gradually introducing the cracks into the specimen. After each static test, the impedance was measured by using 11 identical P-876 DuraAct Patch Transducers [35] of 0.5 mm thickness which were externally bonded with an epoxy adhesive along the FRP strip with a constant spacing of 12 cm (Figure 18). For this, both the impedance signatures and the guided waves were measured under the same conditions explained for the two previous examples. After that, four different loading stages were considered by applying the static loads of 26 (stage D1), 40 (stage D2), 65 (stage D3), and 100 kN (stage D4). Before measuring both the impedances and guided waves, the beam was previously unloaded after each loading stage. The experimental set-up is shown in Figure 19.

### 4.2. Results and Discussion

**Figure 20:**
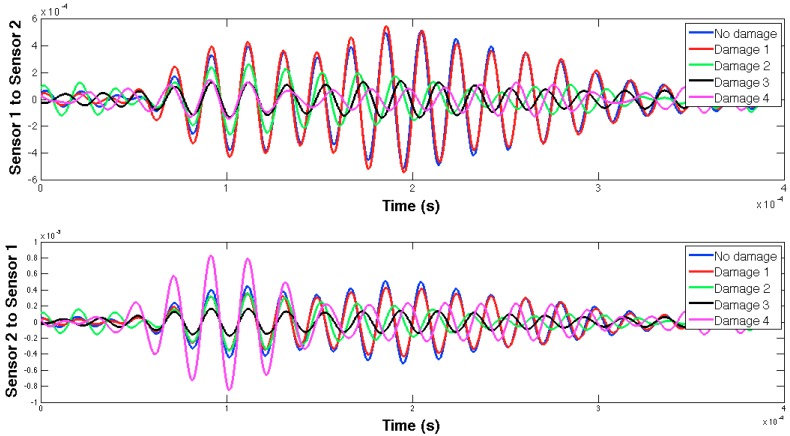
EMPD at 50 kHz. Tone-burst sent and measured between sensors 1 and 2.

**Figure 21:**
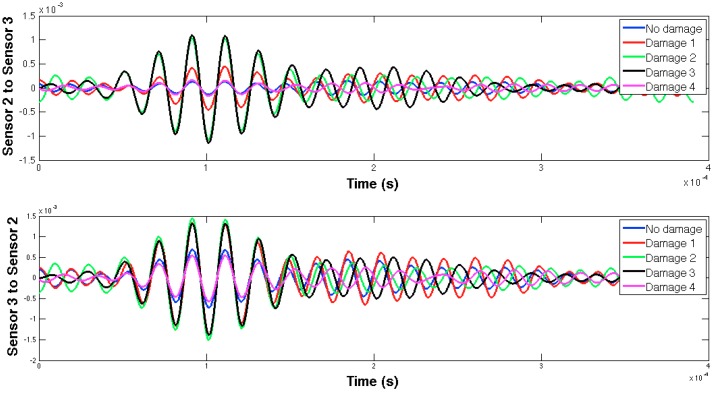
EMPD at 50 kHz. Tone-burst sent and measured between sensors 2 and 3.

**Figure 22:**
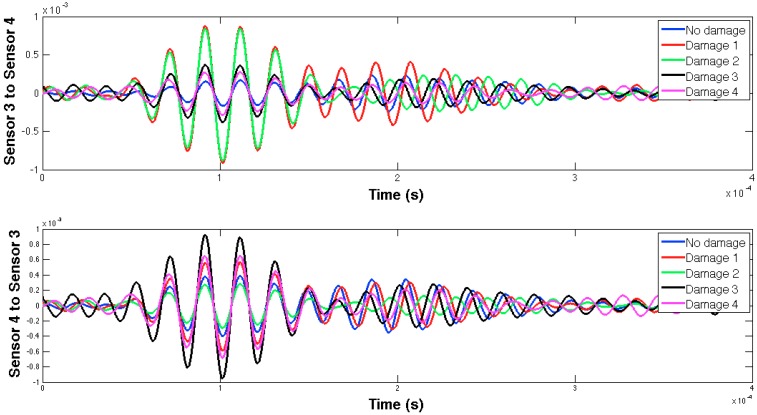
EMPD at 50 kHz. Tone-burst sent and measured between sensors 3 and 4.

**Figure 23:**
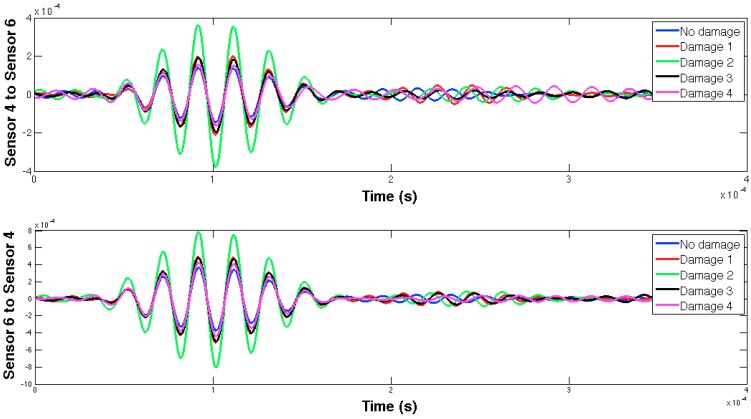
EMPD at 50 kHz. Tone-burst sent and measured between sensors 4 and 6.

**Figure 24:**
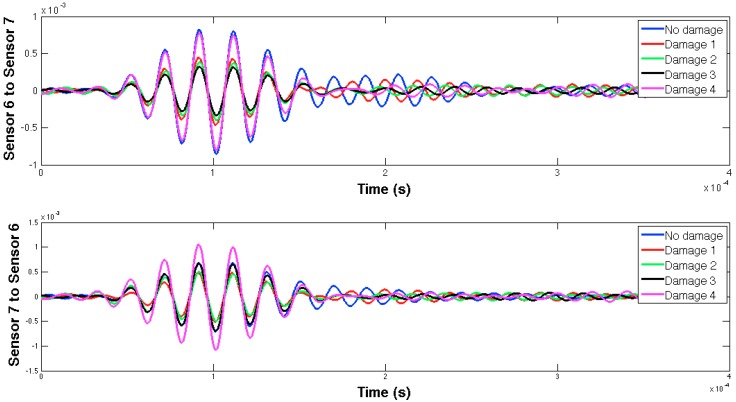
EMPD at 50 kHz. Tone-burst sent and measured between sensors 6 and 7.

**Figure 25:**
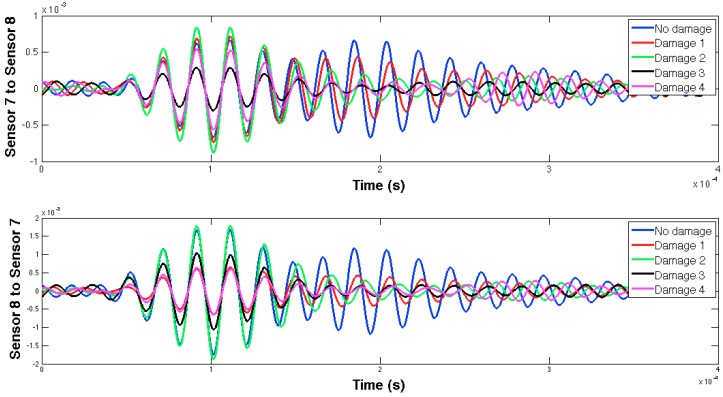
EMPD at 50 kHz. Tone-burst sent and measured between sensors 7 and 8.

**Figure 26:**
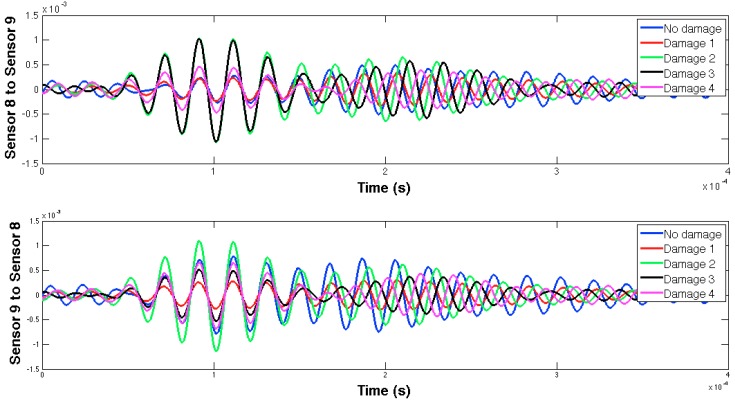
EMPD at 50 kHz. Tone-burst sent and measured between sensors 8 and 9.

**Figure 27:**
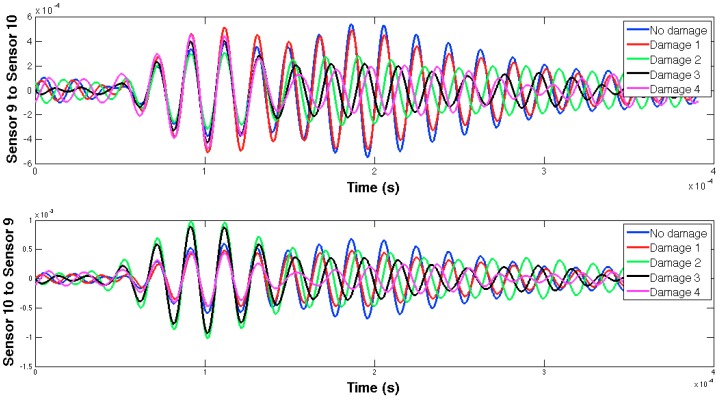
EMPD at 50 kHz. Tone-burst sent and measured between sensors 9 and 10.

The selected guided wave was sent between every two consecutive working sensors, and the EMPD values obtained from all those measurements are shown in Figure 20, Figure 21, Figure 22, Figure 23, Figure 24, Figure 25, Figure 26 and Figure 27. As can be easily verified, the EMPD presents different shapes depending on the sensing region, on the damage scenario, as well as on the direction of motion of the guided waves, as in the two previous examples, which comes to confirm the high sensitivity of this new indicator to the presence of damage, even when dealing with structural systems of such complexity as the one addressed in this section. Furthermore, the differences in the EMPD depending on the direction of the tone-burst is clearer in this case than in the previous two examples, for almost all damage cases.

**Figure 28:**
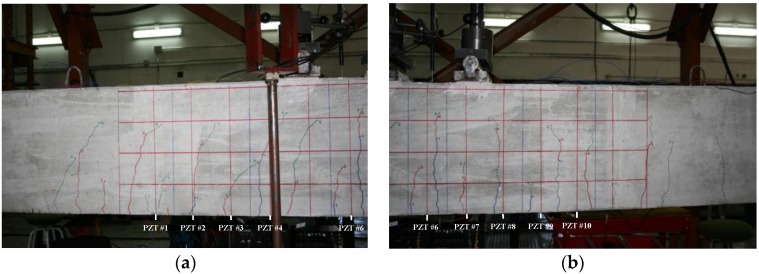
Cracking map of the beam after the last loading stage. (**a**) Left side of the beam; (**b**) right side of the beam.

**Figure 29:**
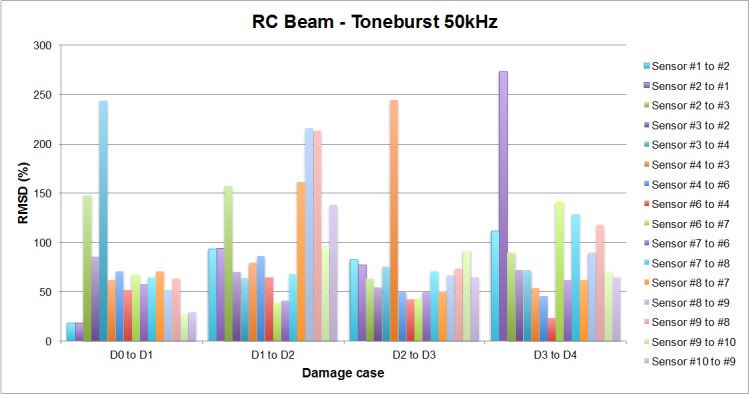
RMSD values for the RC beam.

Each of the graphics shown in Figure 20 demonstrates the presence of the series of damage stages generated in the RC strengthened beam, and it is even possible to discriminate which one could have a higher impact on each sensing region. However, this is just a qualitative analysis of a really complex damage scenario, as shown in Figure 28. Thus, a better understanding will be achieved through the analysis of the RMSD values, as in the previous examples. These values are presented in Figure 29.

**Figure 30:**
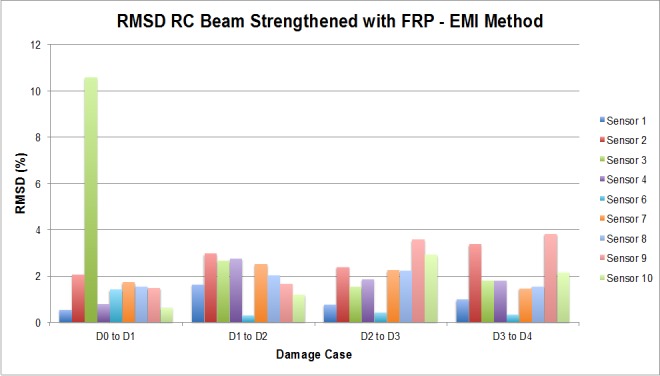
RMSD values for the RC beam corresponding to the EMI method.

From Figure 29, it is quite obvious that every loading step generates a set of damages that influence the calculated value of the EMPD. In this case, just by looking at the RMSD obtained values, the first conclusion is that the EMPD is quite sensitive to all damage present in the structure, regardless of its nature (debonding or flexural cracks). This conclusion can be stated due the fact that for every damage stage, almost all measurements give RMSD values close to or higher than 50%. Furthermore, the most remarkable conclusion corresponds to the first loading step, after which no visible flexural cracks were appreciated on the concrete surface (Figure 28), at least apparently. However, for this damage stage, all measurements clearly detect damage with high RMSD values, which means that there were minor damages generated in the vicinity of the interface between the concrete and the FRP. Hence, thanks to the high sensitivity of this integrated damage detection procedure, it is possible to detect damages at a very early stage of the loading procedure, even in spite of the complexity of the structural system under study. This, however, was not possible to determine so clearly by means of the traditional EMI, whose corresponding RMSD values are shown in Figure 30. In this last figure, all damage indications are much lower than those shown in Figure 29 for all sensors and damage cases, so with this test on a real-scale structure, it is fully demonstrated that the EMPD shows higher potential than the traditional EMI on its own, at least in terms of sensitivity to damage, minor damage in particular. Thanks to this, even small and non-visible incipient flexural cracks can be detected on the structure.

**Figure 31:**
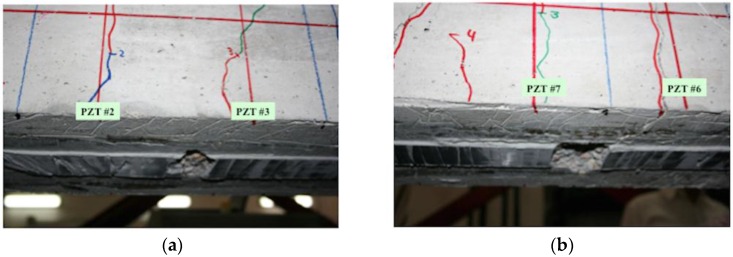
Damages with concrete cover separation between sensors 2 and 3 (**a**) and sensors 6 and 7 (**b**) for the RC beam.

**Figure 32:**
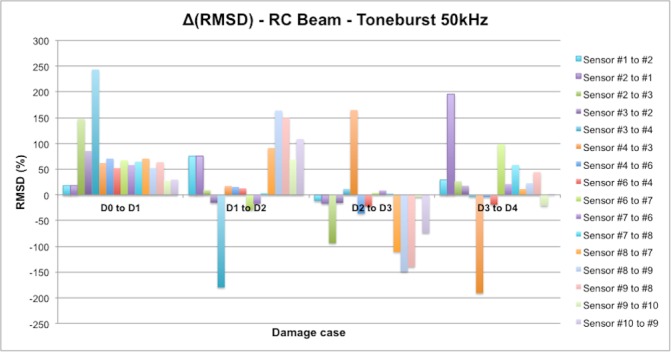
RMSD increments between loading steps for the RC beam corresponding to the EMPD method.

On the other hand, in Figure 29 there are several RMSD values that reach values around 150% and above, clearly far from the rest of the results. This, in the opinion of the authors, could suggest the presence of two different types of damage: while smaller RMSD values would indicate the presence of flexural cracks, these last values would indicate physical damages in the vicinity of the interface between FRP and concrete, which means an origin for debonding appearance. Some of these high values are obtained around sensor 3 for damages D1, D2, and D3 (D1: 147% when the tone-burst is sent from sensor 2 to sensor 3 and 243% from sensor 3 to 4; D2: 156% from sensor 2 to 4; D3: 244% from sensor 4 to 3), suggesting thus the potential presence of debonding in this region of the beam, which comes to be true as can be verified from Figure 31, where the debonding damages after the last loading step until failure are shown. However, this conclusion is apparently not confirmed by any sensor for the second debonding present in the sensing region between sensors 6 and 7. Therefore, further analysis is needed in this region, for which Figure 32 is proposed.

In Figure 31, the RMSD increments from one damage case to the following one is shown, so that a positive value means higher damage severity in the subsequent state, while a negative value means that a lower damage has been induced in the subsequent state. In this sense, just the positive values are important for our purpose, since the interest of this analysis is to find sensing regions likely to suffer a failure mechanism initiated by debonding appearance, which will happen when damage indications grow significantly during the last loading steps. Some high indications are found around sensors 8 and 9 for damage D2, in both Figure 29 and Figure 31, corresponding to two different flexural cracks larger than 15 cm appearing in this region after damage D2. However, none of these indications increase again in the fourth loading step, which suggests that no debonding damage was initiated in this region. The contrary of this happens then in the sensing region between sensors 6 and 7. As can be appreciated in Figure 31, there is an indication slightly higher than 50% after the first loading step (D1), and then the damage is not significantly increased after D2 and D3, but suddenly there is an increase of almost 100% after the last measured loading step, D4, when the EMPD is calculated from measurements taken at sensor 7, which suggests that in this region the interface between concrete and FRP is likely to suffer debonding as well (Figure 32). Although it is true that this debonding is not so obvious as the one detected between sensors 2 and 3, with this methodology it is still possible to analyze each of the sensing regions with a higher level of detail and more accuracy than with the traditional EMI, which is not even capable of offering a clear distinction between stages of damage (Figure 30).

Finally, there is an indication higher than 250%, from sensor 2 to sensor 1 for damage stage D4, which is not related to any debonding either. In this case, as can be seen in Figure 28, a new flexural crack bigger than 10 cm appeared right in the position of sensor 1, which is probably the reason for this unusual RMSD value almost at the end of the loading procedure. Once again, the methodology proposed in this paper enhances the performance of the traditional EMI method, given that this damage is not even noticeable in Figure 30, although there is certainly an important crack generated right in the position of sensor 1, as indicated above.

## 5. Conclusions

A new damage indicator has been proposed in this paper based on Electro-Mechanical Power Dissipation (EMPD), as a result of combining impedance signatures and guided waves into an integrated damage detection procedure. This proposed methodology supposes a new approach in the SHM field, and it has been successfully tested on two different lab-scale structures. The first of these structures was selected mainly in order to prove the sensitivity of the EMPD to the presence of damage, using for that purpose a well-known structure that has been widely used before in the literature. With the second specimen, the goal pursued and achieved in this paper is double. Firstly, thanks to the proposed methodology, guided waves have been used to test more complex structures than what the authors have found in the literature, even though a good number of references suggest that they are not suitable to be used in complex structures. Secondly, the studied concrete specimen is subjected to a more complex series of damage scenarios, involving different heterogeneous materials and complicated behaviours at the interfaces between these materials, and it has been proven how guided waves, through the application of this integrated procedure, can successfully contribute to positive damage identification even for instances of damage as complicated as those studied for this specimen.

Furthermore, some conclusions can also be extracted about damage location, just by paying attention to the direction of motion of the guided wave. In that sense, good results have been presented for single damage scenarios in both lab-scale structures, although the results needs to be improved in the case of multiple damage scenarios.

Finally, the EMPD has also been demonstrated to be sensitive to damage in a real and complex structural system such as the RC beam externally strengthened with FRP. In this case, extremely high sensitivity has been demonstrated even to minor damage at a very early stage of the loading procedure, which means that early detection can be successfully performed.

Hence, this new integrated method implements a promising combination of impedance signatures and guided waves that has been demonstrated to be sensitive to different damages, and which is suitable to work even for real-scale complex structural systems.

## Abbreviations

The following abbreviations are used in this manuscript:

SHM	Structural Health Monitoring
NDT	Non-Destructive Testing
PZT	Lead-Zirconate-Titanate
EMI	Electro-Mechanical Impedance
EMPD	Electro-Mechanical Power Dissipation
FFT	Fast Fourier Transform
IFFT	Inverse Fast Fourier Transform
